# Supplementary material for: Socio-demographic determinants of infectious disease-related health literacy and knowledge in Armenia: Results from a nationwide survey
Source: PLoS One. 2024 Jul 18;19(7):e0307300. doi: 10.1371/journal.pone.0307300 (PMC11257219; doi:10.1371/journal.pone.0307300)
Supplement: Attachment — Submitted filename: Response to Reviewers.docx [file pone.0307300.s001.docx]

**Dear Editorial Team,**

Thank you for the review and the constructive feedback on our manuscript entitled “Socio-demographic determinants of infectious disease-related health literacy and knowledge in Armenia”.

On behalf of the authors’ team, I would like to upload the revised version of the manuscript which addresses the reviewer’s comment. Please find our response below.

Journal requirements:

1. When submitting your revision, we need you to address these additional requirements.

Please ensure that your manuscript meets PLOS ONE's style requirements, including those for file naming. The PLOS ONE style templates can be found at 
<https://journals.plos.org/plosone/s/file?id=wjVg/PLOSOne_formatting_sample_main_body.pdf> and 
<https://journals.plos.org/plosone/s/file?id=ba62/PLOSOne_formatting_sample_title_authors_affiliations.pdf>.

*Thank you, we followed the guidelines while preparing the revisions.*

2. Thank you for stating the following financial disclosure: 
 [Support to control covid-19 and other infectious disease outbreaks. Cooperative Agreement # 72011120CA00003. United States Agency for International Development (USAID)].  
Please state what role the funders took in the study.  If the funders had no role, please state: ""The funders had no role in study design, data collection and analysis, decision to publish, or preparation of the manuscript."" 
If this statement is not correct you must amend it as needed. 
Please include this amended Role of Funder statement in your cover letter; we will change the online submission form on your behalf.

*Support to control COVID-19 and other infectious disease outbreaks. Cooperative Agreement # 72011120CA00003. United States Agency for International Development (USAID).*

*“This study is made possible by the generous support of the American People through the United States Agency for International Development (USAID). The contents of this paper are the sole responsibility of the American University of Armenia Fund and do not necessarily reflect the views of USAID or the United States Government.”*

3. We note that you have indicated that there are restrictions to data sharing for this study. PLOS only allows data to be available upon request if there are legal or ethical restrictions on sharing data publicly. For more information on unacceptable data access restrictions, please see <http://journals.plos.org/plosone/s/data-availability#loc-unacceptable-data-access-restrictions>.

Before we proceed with your manuscript, please address the following prompts:

a) If there are ethical or legal restrictions on sharing a de-identified data set, please explain them in detail (e.g., data contain potentially identifying or sensitive patient information, data are owned by a third-party organization, etc.) and who has imposed them (e.g., a Research Ethics Committee or Institutional Review Board, etc.). Please also provide contact information for a data access committee, ethics committee, or other institutional body to which data requests may be sent.

b) If there are no restrictions, please upload the minimal anonymized data set necessary to replicate your study findings to a stable, public repository and provide us with the relevant URLs, DOIs, or accession numbers. For a list of recommended repositories, please see
<https://journals.plos.org/plosone/s/recommended-repositories>. You also have the option of uploading the data as Supporting Information files, but we would recommend depositing data directly to a data repository if possible.

We will update your Data Availability statement on your behalf to reflect the information you provide.

*We made the underlying data available. The minimal de-identified data is publicly available here:* [*DOI 10.17605/OSF.IO/TW2AX*](https://osf.io/TW2AX/)

**Reviewer #1:**

Dear Authors,
This article deals with a very interesting topic. The relevance of this article is undeniable. Additionally, the article is well structured, the methods of analysis are relevant, and the tables are informative. However, before its publication, some changes need to be made.

You will find my comments below.

Sincerely yours
----------------------------------------------

*Thank you!*

1. The introduction needs polishing. The authors should add statistics on infectious diseases in Armenia.

*We polished the introduction section and added statistics on most common infectious diseases in Armenia. (lines 38-103)*

2. The authors mention that ‘The study team conducted a phone survey in the scope of existing larger study of COVID-19…’. What is the objectives of this phone survey ?

*We added the primary objective of the larger study.*

*“The primary objective of the study was to estimate the seroprevalence of antibodies against SARS-CoV-2 and assess the population’s knowledge, attitude, and practices regarding infectious diseases transmission, manifestation, and treatment in general among adults in Armenia.” (lines 107-110)*

3. The authors said that ‘The infectious disease knowledge was measured using four questions.’ I suggest them to add the four questions.

*We added the four questions in the methods section. (lines 151-155)*

4. ‘The infectious disease-specific health literacy was measured using nine questions’. Same comment as above.

*We added the nine questions. (lines 160-175)*

5. In the discussion section do not use ‘It seems’. Use other words. ‘It seems’ is not a scientific word in these sentences.

*Thank you for the comment. We replaced the word “it seems” with “it is likely” (line 264) and removed word “seem” (line 256).*

6. I suggest to authors to refine the discussion. For example, to explain the effect of age, you need to take into account the differences in educational levels between younger and older people. You need to mobilize data and other studies on Armenia's socio-cultural context.

*We added “This may be due to a lower education levels in older adults, although a majority (70.5%) of our sample had at least a university or vocational degree.” (lines 273-277)*

*7. Finally, The authors need to add limitations regarding the type of survey (phone survey). In addition, I suggest that the authors add a section ‘P*olicy implication of findings’

*We would like to explain why the type of the survey being a phone survey might not be considered a limitation for this study. Out of 3,727 people who underwent blood sampling for the antibody testing 3,483 (93.5%) participated in the phone survey. The study participants gave written informed consent that after the blood sampling they will be contacted by the research team for a phone survey. However, it might be that those who agreed to give a blood sample for the bigger study might be more concerned and aware of their health and had a higher degree of health literacy and knowledge compared to those who refused to participate. To acknowledge this, we added in the limitations that “Also, those more concerned about their health and/or more aware of infectious diseases might agree to participate in the blood sampling for COVID-19 antibody testing introducing some degree of self-selection bias”. (lines 299-301) We also added a section on the Policy implications. (lines 311-328)*

**Reviewer #2:**

Employing a comprehensive nationwide phone survey, the study endeavours to measure the extent of health literacy and knowledge concerning infectious diseases across Armenia, while also examining the impact of socio-economic factors on these variables.
The article is generally well-written, and it includes a thorough review of the literature, presents a sound methodology, and discusses the findings in a comprehensive manner.
Some minor revisions can be addressed to enhance the credibility and interpretability of the research findings; details are reported below.

*Thank you!*

1. I believe that an evidence-based definition of “working from home” and a consistent use of the related terminology for this exposure.

*We would like to explain why we have used “stay at home from work/school” in some infectious disease-related literacy questions in our instrument. The questions were taken from the WHO survey tool designed to guide behavioural insights studies related to COVID-19 (*[*https://iris.who.int/bitstream/handle/10665/333549/WHO-EURO-2020-696-40431-54222-eng.pdf?sequence=1&isAllowed=y*](https://iris.who.int/bitstream/handle/10665/333549/WHO-EURO-2020-696-40431-54222-eng.pdf?sequence=1&isAllowed=y)*). We did not change the wording as the similar wording was used by the government (Ministry of Health) to communicate the COVID-19 related preventive recommendations. We used the same terminology/wording, so the questions are understandable to our survey participants representing general population of Armenia.*

2. The study design should be understood from the title and the abstract; it should bel also mentioned in the methods section.

*We added the study design in the title:* *“Socio-demographic determinants of infectious disease-related health literacy and knowledge in Armenia: Results from a nationwide survey”. (Lines 1-3)*

3. Even though the language adopted in the article is correct, I suggest refining the style to achieve better clarity and precision. Be careful in ensuring that the terminology used is the most accurate and specific to avoid ambiguity.

*We edited throughout the manuscript to better refine the style and clarity.*

4. The methodology is well-structured, employing a nationwide phone survey to collect data. However, the study could benefit from a more detailed description of the survey questions and the rationale behind the selection of these specific questions. Clarification on how the questions were tailored to assess health literacy and knowledge specifically related to infectious diseases would strengthen the paper.

*We added in the Data collection and study tool section explanation.* *“The infections disease-related health literacy questions were adapted to from the WHO survey tool designed to guide behavioural insights studies related to COVID-19 to cover general infectious disease-related health literacy.” (Lines 151-159)*

5. It's mentioned that the scale used to assess infectious disease-related health literacy was not previously validated, which could affect the reliability of the results. If possible, use a validate scale or, in future studies, consider validating the scale used in this paper.

*Thank you for this note. Given that there was (is) no validated tool available in Armenian language, the infections disease-related health literacy questions were taken from the WHO survey tool designed to guide behavioral insights studies related to COVID-19. However, we adapted the scale to cover not only COVID-19 but infectious disease literacy in general and translated to Armenian.*

6. While the paper acknowledges several limitations, including the potential bias introduced by phone surveys and the lack of validation for the health literacy scale, a more thorough exploration of these limitations and their implications for the study's findings would be beneficial. Additionally, discussing potential strategies for overcoming these limitations in future research could provide a more comprehensive picture.

*We added on the recommendations for future similar studies "Future studies may consider using validated scales and designing comprehensive questionnaires specifically focusing on the infectious disease-related health literacy and knowledge" (Lines 306-307)*

7. A detailed participant flowchart or description of participant recruitment, inclusion, and exclusion criteria would enhance transparency in reporting.

*We added a reference to a study which explains the participant recruitment, inclusion, and exclusion criteria in more details (lines 125-126).*

8. The discussion effectively highlights the importance of the study findings in the context of Armenia and beyond. However, the paper would benefit from a broader consideration of the implications for public health policy and education strategies. Specifically, recommendations for how policymakers and educators can use these findings to improve health literacy and preparedness for future infectious disease outbreaks would be useful.

*We added a section on the Policy implications. (lines 311-328)*
